# Quantitative Influence of ABO Blood Groups on Factor VIII and Its Ratio to von Willebrand Factor, Novel Observations from an ARIC Study of 11,673 Subjects

**DOI:** 10.1371/journal.pone.0132626

**Published:** 2015-08-05

**Authors:** Jaewoo Song, Fengju Chen, Marco Campos, Doug Bolgiano, Katie Houck, Lloyd E. Chambless, Kenneth K. Wu, Aaron R. Folsom, David Couper, Eric Boerwinkle, Jing-fei Dong

**Affiliations:** 1 Puget Sound Blood Center Research Institute, Puget Sound Blood Center, Seattle, Washington, United States of America; 2 Department of Laboratory Medicine, Yonsei University, College of Medicine, Seoul, Korea; 3 Human Genetic Center, University of Texas School of Public Health, Houston, Texas, United States of America; 4 Cardiology Section, Department of Medicine, Baylor College of Medicine, Houston, Texas, United States of America; 5 Department of Biostatistics, University of North Carolina, Chapel Hill, North Carolina, United States of America; 6 National Health Research Institutes, Taipei, Taiwan; 7 Division of Epidemiology & Community Health, School of Public Health, University of Minnesota, Minneapolis, Minnesota, United States of America; 8 Division of Hematology, Department of Medicine, University of Washington, School of Medicine, Seattle, Washington, United States of America; National Cerebral and Cardiovascular Center, JAPAN

## Abstract

ABO blood groups are known to influence the plasma level of von Willebrand factor (VWF), but little is known about the relationship between ABO and coagulation factor VIII (FVIII). We analyzed the influence of ABO genotypes on VWF antigen, FVIII activity, and their quantitative relationship in 11,673 participants in the Atherosclerosis Risk in Communities (ARIC) study. VWF, FVIII, and FVIII/VWF levels varied significantly among O, A (A1 and A2), B and AB subjects, and the extent of which varied between Americans of European (EA) and African (AA) descent. We validated a strong influence of ABO blood type on VWF levels (15.2%), but also detected a direct ABO influence on FVIII activity (0.6%) and FVIII/VWF ratio (3.8%) after adjustment for VWF. We determined that FVIII activity changed 0.54% for every 1% change in VWF antigen level. This VWF-FVIII relationship differed between subjects with O and B blood types in EA, AA, and in male, but not female subjects. Variations in FVIII activity were primarily detected at low VWF levels. These new quantitative influences on VWF, FVIII and the FVIII/VWF ratio help understand how ABO genotypes differentially influence VWF, FVIII and their ratio, particularly in racial and gender specific manners.

## Introduction

ABO blood group antigens present on red blood cells are an important determinant of transfusion compatibility. By modifying carbohydrate side chains of soluble and membrane-bound proteins, they are also involved in physiologic and pathologic processes. ABO blood types have been associated with the development of coronary heart disease, stroke, and venous thromboembolism [1–3], in part by regulating levels of von Willebrand factor (VWF) and coagulation factor VIII (FVIII) in the circulation [4;5]. A recent genome wide association study by the CHARGE consortium shows that single nucleotide polymorphisms (SNPs) with the strongest association with VWF levels and FVIII activity are in the ABO locus [4].

VWF is synthesized as a pre-pro-polypeptide monomer of 2,813 amino acids [6]. Each VWF monomer contains 12 potential N- glycosylation and 10 O-glycosylation sites [7], some that are modified by ABO determinants [8;9]. ABO was originally detected on N-linked glycans of VWF [8–10], accounting for up to 13% of the N-linked oligosaccharide chains of VWF multimers in the circulation [8;11]. In addition, a recent study also identified 3 O-linked glycans on VWF that are modified by ABO [12]. The life-span of circulating VWF is known to be influenced by ABO [5;13–16].

Although impacts of ABO on plasma levels and adhesive activity of VWF are well documented, critically important questions remain. First, is the impact of ABO on levels of VWF antigen modified by gender and race? Second, is there a direct ABO influence on FVIII activity that is independent of VWF? A relatively weak VWF-independent impact of ABO on FVIII has previously been reported [17;18], but the size and racial differences of this effect are unknown. Without defining this VWF independent effect, it is difficult to answer the question of whether ABO has a direct or indirect effect on the FVIII/VWF ratio, which is widely used to evaluate VWF synthesis and its defects in clinical settings [17]. Finally, is the ABO effect modified by environmental factors known to affect plasma VWF? We have analyzed data from the Atherosclerosis Risk in Communities (ARIC) study [19–21] to answer these questions. We have previously used this database to determine that ABO accounts for 15.4% and 10.7% of the variability of plasma VWF antigen and FVIII activity in a cohort of 10,434 subjects of European (EA) and African (AA) descents [19;20]. Here, we present data from a cross-sectional study of 11,673 ARIC subjects designed to examine 1) VWF-dependent and independent influences of ABO on FVIII activity and the FVIII-VWF ratio and 2) gender, racial, and environmental modifications of these influences.

## Materials and Methods

### Study population and baseline measurements

ARIC (*www.cscc.unc.edu/aric/*) is a prospective epidemiologic study conducted in four

US communities, designed to investigate the etiology and natural history of atherosclerosis and clinical atherosclerotic diseases, as well as variation in cardiovascular risk factors, medical care, and disease by race, gender, location, and date [22;23]. To examine the intrinsic effects of ABO on VWF and FVIII, adjustments were made for covariates known to affect VWF antigen and FVIII activity in the circulation; including age, race, gender, body mass index (BMI), hypertension, diabetes and history of smoking [24;25]. The use of the ARIC data was approved by the institutional review boards of all participating institutions for the ARIC study.

VWF antigen levels and FVIII activity were measured as previously described [19;20]. The reliability coefficient (1 –intra-individual variance/total variance) obtained from repeat testing of individuals over several weeks was 0.68 for VWF and 0.86 for FVIII.

### ABO genotyping

ABO was genotyped using two SNPs: RS8176719 and RS8176746 (S1 Table). Subjects were classified as type O (OO), type A (AA or AO), type B (BB or BO) or type AB (AB) using these two SNPs. The A subjects were further genotyped as A1 (including A1O), A1A2, and A2 (including A2O) using haplotypes from the ABO SNPs rs8176749, rs8176704 and rs687289 [26].

### Statistical analysis

Distributions of VWF levels and FVIII activity were evaluated to assess normality. Since distributions of the two factors were highly skewed, a natural log transformation was performed prior to regression analysis. The simple linear and multiple linear regression models and the least square regression plots were used to investigate relations among VWF levels, FVIII activity, and the FVIII/VWF ratio. Omega squared (ω^2^) and semipartial ω^2^ were calculated to evaluate effect sizes of the model and individual factors on FVIII activity and FVIII/VWF ratio, respectively. For assessing difference between groups, analysis of variance (ANOVA) or analysis of covariance (ANCOVA) was performed. Multiple comparisons were corrected by the Bonferroni method and p < 0.0045 was considered to be statistically significant. All analyses were performed using SAS 9.2 (SAS Institute, Cary, NC) or R 2.13.2.

## Results

### Baseline characteristics

A total of 11,673 ARIC subjects were included in this cross-sectional study, comprised of 8,817 EA and 2,856 AA subjects. The features and potential confounding characteristics of this demographic are summarized in Table 1. The distribution of ABO blood groups was consistent with previous reports [24]. Among covariates known to affect VWF levels in the circulation, diabetes and hypertension were more common in subjects with type B blood. The B type subjects also had the highest BMI.

**Table 1:** Baseline Characteristics of Subjects in Each ABO Blood Group^‡^.

	ABO blood group							
Characteristic	Total	O	A1	A1A2	A2	B	AB	P value
N	11,673							
^†^Age: mean (SD)	54.0 (5.7)	53.9 (5.7)	54.2 (5.7)	54.2 (5.6)	54.0 (5.7)	54.0 (5.8)	53.7 (5.8)	0.41*
AA Female	1,787	901 (50.4%)	320 (17.9%)	17 (1.0%)	150 (8.4%)	328 (18.4%)	71 (4.0%)	
AA Male	1,069	558 (52.2%)	157 (14.7%)	11 (1.0%)	67 (6.3%)	230 (21.5%)	46 (3.0%)	
EA Female	4,688	1,880 (40.1%)	1,644 (35.1%)	131 (2.8%)	401 (8.6%)	443 (9.4%)	189 (4.0%)	
EA Male	4,129	1,666 (40.3%)	1,358 (32.9%)	130 (3.1%)	407 (9.9%	397 (9.6%)	171 (4.1%)	
BMI: mean (SD)	27.6 (5.3)	27.8 (5.4)	27.2 (5.0)	27.3 (4.8)	27.5 (4.8)	28.0 (5.6)	27.8 (5.5)	<0.0001*
Ever smoking	6,810 (58.4%)	2,874 (57.5%)	2,036 (58.5%)	188 (65.1%)	617 (60.4%)	807 (57.8%)	288 (60.4%)	0.08**
Hypertension	3,907 (33.6%)	1,730 (34.8%)	1,076 (31.1%)	78 (27.1%)	330 (32.4%)	542 (38.9%)	151 (31.9%	<0.0001**
Diabetes	1,281 (11.0%)	548 (11.0%)	345 (9.9%)	31 (10.7%)	101 (9.9%)	204 (14.7%)	52 (10.9%)	0.0002**

^‡^11,673 subjects were included in the study, but for covariates the numbers of subjects were 11,661 for BMI

^†^ Age at baseline visit

* Analysis of variance

** Chi-square test

### Influence of ABO on VWF and FVIII

VWF levels were the highest in subjects with either B or AB blood group, whereas they were the lowest in O subjects in all four gender-by-race groups (Table 2). The difference in VWF antigen levels among the six ABO blood groups remained statistically significant after adjustment for environmental covariates (Table 2, second rows). The overall difference in the mean VWF antigen between type O subjects and those with B blood group was 31.7%. This difference was significantly greater for AA subjects (32.3% and 32.5% for females and males, respectively) than for EA subjects (29.8% and 29.1% for females and males, respectively, P < 0.001). Although genotyped as A and B subjects, VWF antigen levels were 123 ± 45% and 135 ± 46% for AO and BO subjects, significantly lower than those with homozygous for A (144 ± 52%, *p*<0.0001) and B alleles (160 ± 53%, *p* = 0.0042), respectively. Furthermore, VWF levels differed significantly among A1, A1A2, and A2 genotypes overall and for all 8 gender-by-race groups (p < 0.0001).

**Table 2:** Geometric Mean (95% CI) of VWF Level (%) in ABO Blood Groups*.

Stratum	O	A1	A1A2	A2	B	AB	P value
Overall	92.2 (91.3, 93.1)	124.6 (123.1, 126)	119.8 (115.0, 124.7)	119.8 (115.0, 124.7)	137 (134.5, 139.6)	132.3 (128.2, 136.5)	<0.0001
	95.2 (94.2, 96.1)	132.1 (130.5, 133.8)	127.6 (122.7, 132.6)	100.1 (98.1, 102.2)	138.3 (135.9, 140.8)	138.0 (133.9, 142.2)	<0.0001
AA	103.5 (101.6, 105.5)	144.7 (139.9, 149.6)	144.6 (126.0, 166.1)	111.8 (106.4, 117.5)	156.5 (151.7, 161.4)	156.3 (146.1, 167.2)	<0.0001
	103.4 (101.5, 105.4)	143.1 (138.5, 148.0)	145.7 (127.3, 166.7)	111.2 (105.8, 116.8)	155.8 (151.1, 160.7)	155.5 (145.4, 166.3)	<0.0001
EA	87.9 (86.9, 88.8)	121.6 (120.2, 123.1)	117.4 (112.8, 122.2)	91.7 (89.6, 93.8)	125.5 (122.7, 128.3)	125.3 (121.1, 129.7)	<0.0001
	87.8 (86.9, 88.8)	121.7 (120.3, 123.1)	116.8 (112.3, 121.4)	91.7 (89.7, 93.8)	124.9 (122.2, 127.6)	125.8 (121.7, 130.0)	<0.0001
Female	91.8 (90.6, 93.0)	124.3 (122.4, 126.2)	122.9 (116.1, 130.1)	95.7 (92.9, 98.6)	137.9 (134.5, 141.4)	132.7 (127.1, 138.5)	<0.0001
	94.4 (93.3, 95.7)	131.8 (129.7, 133.9)	131.6 (124.8, 138.9)	99.2 (96.5, 102.0)	139.0 (135.8, 142.3)	137.6 (132.2, 143.3)	<0.0001
Male	92.7 (91.3, 94.0)	124.9 (122.7, 127.1)	116.6 (110.1, 123.5)	95.5 (92.6, 98.6)	136.0 (132.3, 139.7)	131.8 (125.9, 138.1)	<0.0001
	96.1 (94.7, 97.6)	132.4 (129.9, 135.0)	123.7 (117.0, 130.8)	101.3 (98.2, 104.5)	137.5 (133.9, 141.1)	138.5 (132.4, 144.9)	<0.0001
AA female	104.7 (102.1, 107.2)	147.1 (141.2, 153.2)	149.7 (125.4, 178.8)	112.5 (106.0, 119.5)	158.5 (152.2, 165.1)	159.2 (146.0, 173.7)	<0.0001
	105.0 (102.6, 107.6)	145.5 (139.8, 151.4)	153.1 (128.8, 182.0)	112.8 (106.4, 119.7)	159.3 (153.1, 165.7)	157.1 (144.2, 171.2)	<0.0001
AA male	101.7 (98.6, 104.9)	140.0 (132.1, 148.4)	137.1 (110.0, 170.9)	110.2 (100.8, 120.5)	151.9 (136.3, 169.1)	153.6 (146.4, 161.2)	<0.0001
	101.9 (98.8, 105.0)	140.6 (132.7, 149.0)	136.3 (109.7, 169.3)	110.1 (100.8, 120.2)	151.8 (144.7, 159.3)	153.9 (138.2, 171.3)	<0.0001
EA female	86.2 (84.9, 87.5)	120.3 (118.4, 122.2)	119.8 (113.2, 126.7)	90.1 (87.2, 93.1)	124.4 (120.6, 128.3)	123.9 (118.2, 129.9)	<0.0001
	86.3 (85.0, 87.5)	120.4 (118.5, 122.2)	119.1 (112.9, 125.7)	89.7 (87.0, 92.5)	124.1 (120.5, 127.7)	124.4 (118.9, 130.1)	<0.0001
EA male	89.8 (88.4, 91.3)	123.3 (121.1, 125.5)	115.0 (108.6, 121.8)	93.3 (90.3, 96.4)	126.7 (122.6, 131.0)	126.9 (120.7, 133.4)	<0.0001
	89.6 (88.2, 91.0)	123.2 (121.1, 125.3)	114.8 (108.6, 121.3)	93.9 (91.0, 96.9)	125.8 (121.9, 129.9)	127.6 (121.5, 133.9)	<0.0001

* For all strata, top rows: unadjusted values and bottom rows: values adjusted for age, smoking, BMI, diabetes, and hypertension (race and gender were also included for overall population; race in the gender-specific analyses and gender in the race-specific analyses)

Consistent with VWF distribution, FVIII activity was also the lowest in subjects with blood group O and highest in those with either B or AB before and after adjustment for environmental factors (Table 3). Overall, type O subjects had a mean FVIII activity that was 76.7% of those with blood type B, and further analyses indicate a significant racial, but not gender dependent difference between O and B subjects (p < 0.001). The overall difference between the highest and lowest FVIII activity (O vs. B or AB) was reduced from 23.3% to 6.2% after adjustment for VWF (Table 3, second rows). This reduction was similarly observed in all four gender-by-race groups. FVIII activity was significantly different among subjects with A1, A1A2, and A2 genotypes, but further gender-by-race group analyses found that the difference was found only in EA, but not in AA subjects of both genders after the values were adjusted for not only environmental factors, but also for VWF (Table 4).

**Table 3:** Geometric Mean (95% CI) of FVIII Activity (%) in ABO Blood Groups*.

Stratum	O	A1	A1A2	A2	B	AB	P value
	112.4 (111.6, 113.3)	136.1 (134.9, 137.2)	130.7 (126.8, 134.8)	115.8 (113.9, 117.6)	146.5 (144.5, 148.6)	142.4 (139.1, 145.8)	<0.0001
Overall	115.2 (114.4, 116.0)	142.7 (141.5, 144.0)	138.1 (134.3, 142.1)	120.3 (118.5, 122.1)	147.0 (145.1, 148.9)	147.4 (144.2, 150.7)	<0.0001
	122.5 (121.8, 123.2)	129.8 (128.9, 130.8)	127.7 (125.0, 130.5)	124.9 (123.4, 126.3)	130.8 (129.5, 132.2)	131.3 (129.1, 133.5)	<0.0001
	125.1 (123.3, 126.9)	156.2 (152.4, 160.1)	154.7 (139.6, 171.5)	129.8 (125.1, 134.7)	165.1 (161.3, 168.9)	167.6 (159.4, 176.2)	<0.0001
AA	124.8 (123.1, 126.5)	154.2 (150.5, 158.0)	154.6 (140.2, 170.5)	129.1 (124.6, 133.8)	164.1 (160.5, 167.8)	167.0 (159.1, 175.3)	<0.0001
	135.4 (134.0, 136.8)	142.3 (139.8, 144.8)	141.4 (131.7, 151.8)	135.1 (131.7, 138.7)	145.2 (142.7, 147.6)	147.9 (142.7, 153.2)	<0.0001
	107.6 (106.8, 108.5)	133.1 (131.9, 134.3)	128.4 (124.6, 132.3)	112.3 (110.4, 114.2)	135.4 (133.1, 137.6)	135.1 (131.7, 138.6)	<0.0001
EA	107.4 (106.6, 108.2)	132.9 (131.8, 134.0)	128.0 (124.5, 131.7)	112.2 (110.4, 114.1)	134.6 (132.4, 136.7)	135.3 (132.1, 138.6)	<0.0001
	116.4 (115.6, 117.1)	123.6 (122.8, 124.5)	121.5 (118.8, 124.1)	119.2 (117.7, 120.7)	123.7 (122.2, 125.2)	124.0 (121.7, 126.3)	<0.0001
	114.6 (113.5, 115.7)	139.3 (137.7, 140.9)	137.0 (131.3, 142.9)	118.7 (116.1, 121.4)	149.4 (146.6, 152.2)	146.4 (141.8, 151.2)	<0.0001
Female	117.0 (115.9, 118.1)	145.7 (144.1, 147.4)	144.7 (139.2, 150.5)	122.1 (119.6, 124.6)	149.8 (147.2, 152.3)	150.8 (146.4, 155.3)	<0.0001
	124.7 (123.8, 125.7)	132.9 (131.7, 134.1)	132.1 (128.2, 136.1)	127.2 (125.2, 129.2)	133.2 (131.5, 135.0)	134.8 (131.8, 137.9)	<0.0001
	109.8 (108.6, 111.0)	132.0 (130.2, 133.7)	124.5 (119.3, 129.9)	112.4 (109.8, 115.1)	143.1 (140.2, 146.0)	137.8 (133.2, 142.7)	<0.0001
Male	113.5 (112.3, 114.8)	139.7 (137.7, 141.7)	131.8 (126.5, 137.3)	118.6 (115.9, 121.4)	144.3 (141.5, 147.1)	143.9 (139.3, 148.8)	<0.0001
	120.3 (119.3, 121.4)	126.7 (125.3, 128.1)	123.5 (119.8, 127.4)	122.5 (120.5, 124.7)	128.5 (126.6, 130.4)	127.7 (124.6, 131.0)	<0.0001
	127.6 (125.3, 130.0)	160.8 (156.0, 165.8)	157.7 (138.2, 180.0)	131.4 (125.7, 137.4)	168.2 (163.2, 173.3)	172.0 (161.3, 183.5)	<0.0001
AA female	128.2 (126.0, 130.4)	159.0 (154.4, 163.7)	159.3 (140.5, 180.5)	132.0 (126.5, 137.8)	169.9 (159.7, 180.8)	169.0 (164.2, 173.9)	<0.0001
	138.5 (136.6, 140.4)	146.8 (143.6, 150.1)	143.5 (130.7, 157.6)	137.8 (133.5, 142.2)	149.4 (146.1, 152.8)	151.2 (144.3, 158.4)	<0.0001
	121.0 (118.3, 123.8)	147.2 (141.0, 153.6)	150.2 (127.8, 176.6)	126.4 (118.3, 134.9)	160.8 (155.2, 166.6)	161.0 (148.7, 174.2)	<0.0001
AA male	121.3 (118.6, 124.0)	148.4 (142.4, 154.8)	148.8 (127.2, 174.1)	127.0 (119.1, 135.4)	158.7 (153.3, 164.4)	163.6 (151.3, 176.8)	<0.0001
	131.8 (129.7, 133.9)	136.1 (132.2, 140.1)	138.7 (124.5, 154.4)	132.5 (126.8, 138.5)	139.8 (136.3, 143.3)	143.0 (135.5, 150.9)	0.0021
	108.8 (107.6, 110.0)	135.5 (133.9, 137.1)	134.5 (129.0, 140.2)	114.3 (111.6, 117.1)	136.8 (133.7, 139.9)	137.8 (133.1, 142.7)	<0.0001
EA female	108.8 (107.7, 110.0)	135.4 (134.0, 137.0)	134.2 (129.0, 139.5)	114.0 (111.4, 116.5)	136.4 (133.5, 139.3)	138.6 (134.1, 143.2)	<0.0001
	118.2 (117.2, 119.2)	126.1 (125.0, 127.2)	125.5 (121.8, 129.4)	121.5 (119.4, 123.7)	125.2 (123.1, 127.3)	127.1 (123.9, 130.4)	<0.0001
	106.3 (105.0, 107.5)	130.3 (128.6, 132.0)	122.5 (117.4, 127.8)	110.3 (107.7, 112.9)	133.8 (130.6, 137.1)	132.2 (127.4, 137.2)	<0.0001
EA male	106.1 (104.9, 107.3)	130.4 (128.7, 132.0)	122.4 (117.5, 127.5)	110.6 (108.1, 113.2)	132.8 (129.8, 136.0)	132.4 (127.7, 137.2)	<0.0001
	114.7 (113.6, 115.7)	121.3 (120.0, 122.5)	117.7 (114.0, 121.4)	116.9 (114.9, 119.1)	122.3 (120.1, 124.6)	121.1 (117.8, 124.5)	<0.0001

* Top rows: unadjusted values; mid-rows (light grey): adjusted for age, smoking, BMI, diabetes, and hypertension; bottom rows (dark grey): adjusted for model 2 covariates and VWF

**Table 4:** Geometric Mean (95% CI) of FVIII Activity (%) in A Blood Groups*.

Stratum	A1	A1A2	A2	P value
Overall	142.7 (141.5, 144.0)	138.1 (134.3, 142.1)	120.3 (118.5, 122.1)	<0.0001
	129.8 (128.9, 130.8)	127.7 (125.0, 130.5)	124.9 (123.4, 126.3)	<0.0001
AA	154.2 (150.5, 158.0)	154.6 (140.2, 170.5)	129.1 (124.6, 133.8)	<0.0001
	142.3 (139.8, 144.8)	141.4 (131.7, 151.8)	135.1 (131.7, 138.7)	0.010
EA	132.9 (131.8, 134.0)	128.0 (124.5, 131.7)	112.2 (110.4, 114.1)	<0.0001
	123.6 (122.8, 124.5)	121.5 (118.8, 124.1)	119.2 (117.7, 120.7)	<0.0001
Female	145.7 (144.1, 147.4)	144.7 (139.2, 150.5)	122.1 (119.6, 124.6)	<0.0001
	132.9 (131.7, 134.1)	132.1 (128.2, 136.1)	127.2 (125.2, 129.2)	<0.0001
Male	139.7 (137.7, 141.7)	131.8 (126.5, 137.3)	118.6 (115.9, 121.4)	<0.0001
	126.7 (125.3, 128.1)	123.5 (119.8, 127.4)	122.5 (120.5, 124.7)	0.001
AA female	159.0 (154.4, 163.7)	159.3 (140.5, 180.5)	132.0 (126.5, 137.8)	<0.0001
	146.8 (143.6, 150.1)	143.5 (130.7, 157.6)	137.8 (133.5, 142.2)	0.0068
AA male	148.4 (142.4, 154.8)	148.8 (127.2, 174.1)	127.0 (119.1, 135.4)	0.0006
	136.1 (132.2, 140.1)	138.7 (124.5, 154.4)	132.5 (126.8, 138.5)	0.72
EA female	135.4 (134.0, 137.0)	134.2 (129.0, 139.5)	114.0 (111.4, 116.5)	<0.0001
	126.1 (125.0, 127.2)	125.5 (121.8, 129.4)	121.5 (119.4, 123.7)	0.0005
EA male	130.4 (128.7, 132.0)	122.4 (117.5, 127.5)	110.6 (108.1, 113.2)	<0.0001
	121.3 (120.0, 122.5)	117.7 (114.0, 121.4)	116.9 (114.9, 119.1)	0.0008

* **Top row: values adjusted for age, smoking, BMI, diabetes, and hypertension and bottom row (shaded): values adjusted for** environmental factors as in top row, but also for VWF

We have previously shown that ABO contributes to 10.7% of FVIII variability before adjustment for VWF [22], and this study further found that the level of influence was reduced to 0.6% after the VWF adjustment (Table 5, left column). These data are consistent with a strong VWF influence on the variability of FVIII activity in the plasma (quantified to be 35.5%), but also demonstrated a small, but significant VWF-independent influence. We have previously shown that age and BMI contributed to 4.39% and 1.61% of VWF variability [20], but their influences on variability of FVIII activity and FVIII/VWF ratio were minimal (Table 5). So were hypertension, diabetes, and ever smoking status. Race and gender together accounted for 1.15% and 0.83% of variability of FVIII and FVIII/VWF ratio.

**Table 5:** Effect Size (%) of Covariates for FVIII activity and FVIII/VWF ratio.

	Semipartial ω^2^ (%)*	
Predictor	FVIII activity^a^	FVIII/VWF ratio^b^
ABO	0.83	5.00
VWF	30.82	
Age	0.06	1.13
BMI	0.16	0.04
Hypertension	0.08	0.07
Diabetes	0.07	0.04
Ever smoking	0.14	0.37
Race & gender	1.15	0.83

* Semipartial ω^2^ is the proportion of variability explained by each factor.

^a^ Model was defined as log FVIII = log VWF + ABO + environmental covariates (age, BMI, hypertension, diabetes, ever smoking status, and combination of race and gender).

^b^ Model was defined as log FVIII/VWF ratio = ABO + environmental covariates (age, BMI, hypertension, diabetes, ever smoking status, and combination of race and gender).

### Association of ABO with FVIII/VWF ratio

The mean FVIII/VWF ratio was examined among the six ABO blood groups (Table 6). Subjects with O blood group had a significantly higher FVIII/VWF ratio followed by those with A, B, or AB blood group, before and after adjustment for environmental covariates. Similar to FVIII activity, difference among A1, A1A2, and A2 subjects did not reach statistical significance in AA subjects after the Bonferroni correction (data now shown). Quantitatively, ABO contributed 5% of the variability of the FVIII/VWF ratio (Table 5, right column), whereas environmental covariates minimally influenced it.

**Table 6:** Geometric Mean (95% CI) of FVIII/VWF Ratio in ABO Blood Groups*.

Stratum	O	A1	A1A2	A2	B	AB	P value
Overall	1.22 (1.21, 1.23)	1.09 (1.08, 1.10)	1.09 (1.06, 1.12)	1.21 (1.19, 1.23)	1.07 (1.06, 1.08)	1.08 (1.05, 1.10)	<0.0001
	1.21 (1.20, 1.22)	1.08 (1.07, 1.09)	1.08 (1.05, 1.11)	1.20 (1.18, 1.22)	1.06 (1.05, 1.08)	1.07 (1.04, 1.09)	<0.0001
AA	1.21 (1.19, 1.22)	1.08 (1.05, 1.11)	1.07 (0.97, 1.18)	1.16 (1.12, 1.20)	1.06 (1.03, 1.08)	1.07 (1.02, 1.13)	<0.0001
	1.21 (1.19, 1.22)	1.08 (1.05, 1.10)	1.06 (0.96, 1.17)	1.16 (1.12, 1.20)	1.05 (1.03, 1.08)	1.07 (1.02, 1.13)	<0.0001
EA	1.22 (1.21, 1.23)	1.09 (1.08, 1.10)	1.09 (1.06, 1.13)	1.22 (1.20, 1.25)	1.08 (1.06, 1.10)	1.08 (1.05, 1.11)	<0.0001
	1.22 (1.21, 1.23)	1.09 (1.08, 1.10)	1.10 (1.06, 1.13)	1.22 (1.20, 1.24)	1.08 (1.06, 1.10)	1.08 (1.05, 1.10)	<0.0001
Female	1.25 (1.24, 1.26)	1.12 (1.11, 1.13)	1.11 (1.07, 1.16)	1.24 (1.21, 1.27)	1.08 (1.06, 1.10)	1.10 (1.07, 1.14)	<0.0001
	1.24 (1.23, 1.25)	1.11 (1.09, 1.12)	1.10 (1.06, 1.15)	1.23 (1.20, 1.26)	1.08 (1.06, 1.10)	1.10 (1.06, 1.13)	<0.0001
Male	1.18 (1.17, 1.20)	1.06 (1.04, 1.07)	1.07 (1.02, 1.11)	1.18 (1.15, 1.20)	1.05 (1.03, 1.07)	1.05 (1.01, 1.08)	<0.0001
	1.18 (1.17, 1.19)	1.05 (1.04, 1.07)	1.06 (1.02, 1.11)	1.17 (1.14, 1.20)	1.05 (1.03, 1.07)	1.04 (1.00, 1.07)	<0.0001
AA female	1.22 (1.20, 1.24)	1.09 (1.06, 1.13)	1.05 (0.92, 1.20)	1.17 (1.12, 1.22)	1.06 (1.03, 1.09)	1.08 (1.01, 1.15)	<0.0001
	1.22 (1.20, 1.24)	1.09 (1.06, 1.13)	1.04 (0.91, 1.18)	1.17 (1.12, 1.22)	1.06 (1.03, 1.09)	1.08 (1.01, 1.15)	<0.0001
AA male	1.19 (1.17, 1.22)	1.05 (1.01, 1.09)	1.10 (0.94, 1.27)	1.15 (1.08, 1.22)	1.05 (1.01, 1.08)	1.06 (0.99, 1.14)	<0.0001
	1.19 (1.17, 1.22)	1.06 (1.01, 1.10)	1.09 (0.94, 1.27)	1.15 (1.09, 1.23)	1.05 (1.01, 1.08)	1.06 (0.99, 1.14)	<0.0001
EA female	1.26 (1.25, 1.28)	1.13 (1.11, 1.14)	1.12 (1.08, 1.17)	1.27 (1.24, 1.30)	1.10 (1.07, 1.13)	1.11 (1.07, 1.15)	<0.0001
	1.26 (1.25, 1.28)	1.13 (1.11, 1.14)	1.13 (1.08, 1.17)	1.27 (1.24, 1.30)	1.10 (1.07, 1.12)	1.11 (1.08, 1.15)	<0.0001
EA male	1.18 (1.17, 1.20)	1.06 (1.04, 1.07)	1.07 (1.02, 1.11)	1.18 (1.15, 1.21)	1.06 (1.03, 1.08)	1.04 (1.00, 1.08)	<0.0001
	1.18 (1.17, 1.20)	1.06 (1.04, 1.07)	1.07 (1.02, 1.11)	1.18 (1.15, 1.21)	1.04 (1.00, 1.08)	1.06 (1.03, 1.08)	<0.0001

Top row: unadjusted values; bottom row (shaded): values adjusted for age, smoking, BMI, diabetes, and hypertension (race and gender were also included for overall population; race in the gender-specific analyses and gender in the race-specific analyses)

### Interaction between VWF and FVIII

FVIII activity and VWF antigen level correlated well for the entire cohort (S1 Fig) and for four race-by-gender groups (S2 Fig, A1, A1A2, and A2 subjects were analyzed together), with correlation coefficients ranging from 0.57 to 0.79. To delineate a quantitative relation between VWF and FVIII and understand how race and gender may modify this relationship, we plotted log VWF level and log FVIII activity on a coordinated plane. The least-squares trend line suggests an overall linear relationship between the two measurements (R^2^ = 0.54, S1 Fig): there was a 0.54% change in FVIII activity for every 1% change in VWF antigen level. This VWF-FVIII relationship had significantly different slopes between subjects with O and B blood groups for EA, AA, and male, but not female subjects (Table 7). These different slopes resulted in variations in FVIII activity among ABO groups primarily detected at low VWF levels.

**Table 7:** Relationship between log FVIII and log VWF in homozygous ABO types*.

Subject	Slope				Adjusted p value for interaction
	O	A1	A2	B	
Overall	0.484	0.451	0.485	0.448	0.0025
AA	0.515	0.473	0.530	0.458	0.04
EA	0.488	0.447	0.479	0.419	0.001
Female	0.486	0.445	0.481	0.452	0.12
Male	0.509	0.458	0.486	0.442	0.005

* p-values for comparisons between slopes for O and B blood groups.

Models adjusted for age, smoking, BMI, diabetes, and hypertension (race and gender were also included for overall population; race in the gender-specific analyses and gender in the race-specific analyses).

## Discussion

While the influence of ABO on VWF has been extensively studied and widely reported, we have provided quantitative data on the association of ABO with FVIII activity, its modification by VWF, and the FVIII/VWF ratio. The large sample size allowed us to conduct racial and gender subgroup analyses that have not previously been done due to smaller sample sizes. We also examined the quantitative relationship between VWF level and FVIII activity. Consistent with previous reports [24;27], we detected a significant influence of ABO on VWF levels, accounting for 15.2% of overall VWF variability. VWF antigen also differed among A1, A1A2, and A2 subjects. VWF antigen was the lowest in subjects with O blood groups and highest in those with either B or AB blood groups. A significant ABO dose effect on VWF level was detected between AA and AO as well as BB and BO genotypes. In addition to validation of previous findings in smaller samples, we made several novel observations on quantitative impacts on how ABO influenced VWF, FVIII and their ratio.

First, the impact of ABO on VWF has been well documented and reaffirmed recently by the CHARGE Consortium genome-wide association study [4;7;8]. However, whether ABO influences FVIII through VWF-dependent and/or-independent means remains poorly defined. On one hand, Smith NL, *et al [4]* suggested that the transport and chaperoning function of VWF for FVIII was responsible for the association between ABO and FVIII activity. This notion is supported by an earlier study of 158 monozygotic and dizygotic twins where the adjustment for VWF level abolished FVIII differences between ABO blood groups [28]. On the other hand, a VWF-independent effect of ABO on FVIII was detected by studying subjects from control and hemophilic families [18;29]. We found that FVIII activity was highest in samples from blood group AB or B subjects and lowest in subjects with O blood group before and after adjustment for environmental covariates. This distribution was in parallel with the VWF distribution among the six blood groups, but the influence of ABO on FVIII variability was reduced from 10.7% to 0.6% after adjustment for VWF. This suggests that ABO influences FVIII activity primarily, but not exclusively through VWF. The dominant VWF influence was calculated to contribute 30.8% of the FVIII variability (Table 5). The finding also suggests that the ABO modified FVIII, but at a significantly lower level.

Second, the FVIII/VWF ratio is widely used as a means to analyze concordance between FVIII and VWF in the circulation as well as a marker for VWF synthesis [25]. Here, we further quantified the ABO contribution to the variability of the FVIII/VWF ratio to be 5%, which is significantly smaller than the influence of ABO on VWF level (15.2%), but much greater than the impact on FVIII activity (0.6%).

Third, plasma VWF antigen (22%-412%) and FVIII activity (20%-540%) varied significantly among ARIC subjects [19;20]. These large variations are likely due to ABO, intrinsic genetic variability, and environmental factors. Because ABO differentially influences VWF and FVIII, we examined a quantitative relationship between these two factors in each of the four ABO blood groups. FVIII activity was linearly associated with VWF in a logarithmic scale, with every 1% change in VWF resulting in a 0.54% change in FVIII. The slope for this linear relationship differed significantly between O and B blood groups for EA, AA, and male subjects, but not for female subjects. This difference in slopes appears to result in a greater variation of FVIII activity among the four blood groups at low VWF levels (Table 7). Although this population study could not determine whether it is required to maintain a constant molar ratio of the two molecules in the circulation, this relationship defines an intrinsic interaction between FVIII and VWF. It is possible that as a known acute phase reactant, environmental changes contribute more to high VWF levels, whereas ABO is a predominant factor in regulating VWF expression in subjects with a low baseline level of VWF.

In summary, we have determined that the influence of ABO on FVIII activity is primarily mediated by VWF, but a small VWF-independent effect (0.6%) was also detected. ABO contributed to 5% variability of the FVIII/VWF ratio. VWF and FVIII are linearly correlated in logarithmic scale, but the relationship between the two factors varied among the four ABO types. These observations provide quantitative insights into how ABO differentially influences VWF, FVIII and the FVIII/VWF ratio and how race and gender modify these influences. The data also suggest that the influence of ABO on FVIII variability may be greater for subjects who have low baseline levels of VWF.

## Supporting Information

S1 Fig Relationship between VWF and FVIII for the entire cohort samples: (A) the data after adjustment for environmental factors were analyzed using a regression model and (B) the least square trend line was plotted for the entire cohort samples. (DOCX)

S2 Fig Plots for ABO blood groups in each gender and race group presented with regression line. (DOCX)

S1 Table SNP Used for Genotyping ABO Blood Groups. (DOCX)
