# Supplementary material for: Quantitative Influence of ABO Blood Groups on Factor VIII and Its Ratio to von Willebrand Factor, Novel Observations from an ARIC Study of 11,673 Subjects
Source: PLoS One. 2015 Aug 5;10(8):e0132626. doi: 10.1371/journal.pone.0132626 (PMC4526567; doi:10.1371/journal.pone.0132626)
Supplement: S1 Fig — (DOCX) [file pone.0132626.s001.docx]

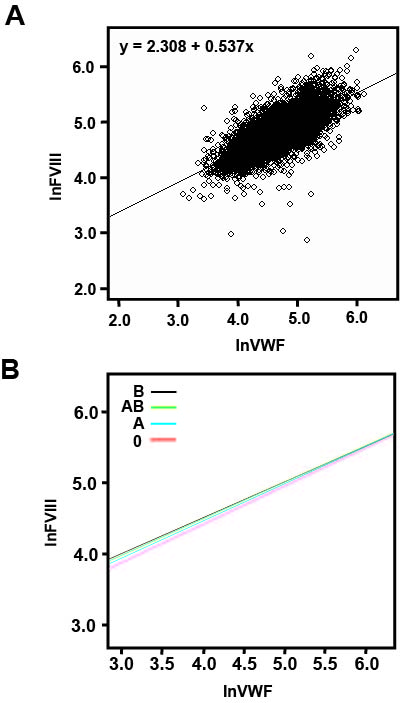


**Figure S1: Relationship between VWF and FVIII for the entire cohort samples. (A)** The data after adjustment for environmental factors were analyzed using a regression model. **(B)** The least square trend line was plotted for the entire cohort samples.
