# Supplementary material for: Quantitative Influence of ABO Blood Groups on Factor VIII and Its Ratio to von Willebrand Factor, Novel Observations from an ARIC Study of 11,673 Subjects
Source: PLoS One. 2015 Aug 5;10(8):e0132626. doi: 10.1371/journal.pone.0132626 (PMC4526567; doi:10.1371/journal.pone.0132626)
Supplement: S2 Fig — (DOCX) [file pone.0132626.s002.docx]

**S-Figure 2:** Plots for ABO blood groups in each gender and race group presented with regression line.
