# Supplementary material for: Quantitative Influence of ABO Blood Groups on Factor VIII and Its Ratio to von Willebrand Factor, Novel Observations from an ARIC Study of 11,673 Subjects
Source: PLoS One. 2015 Aug 5;10(8):e0132626. doi: 10.1371/journal.pone.0132626 (PMC4526567; doi:10.1371/journal.pone.0132626)
Supplement: S1 Table — (DOCX) [file pone.0132626.s003.docx]

**Table S1: SNP Used for Genotyping ABO Blood Groups**

| **ABO** | **RS8176719** | **RS8176746** |
| --- | --- | --- |
| AA | GG | GG |
| AB | GG | GT |
| BB | GG | TT |
| AO | -G | GG |
| BO | -G | TT |
| BO | -G | GT |
| OO | -- | n/a |
